# Impact of the COVID-19 Pandemic on Melanoma Diagnosis in Romania—Data from Two University Centers

**DOI:** 10.3390/ijerph192215129

**Published:** 2022-11-16

**Authors:** Loredana Ungureanu, Adina Patricia Apostu, Ștefan Cristian Vesa, Alexandra Elena Cășeriu, Simona Frățilă, Gabriela Iancu, Nona Bejinariu, Maximilian Munteanu, Simona C. Șenilă, Alina Vasilovici

**Affiliations:** 1 Department of Dermatology, “Iuliu Hațieganu” University of Medicine and Pharmacy, 400006 Cluj-Napoca, Romania; 2 Department of Dermatology, Emergency County Hospital, 400006 Cluj-Napoca, Romania; 3 Department of Pharmacology, Toxicology and Clinical Pharmacology, “Iuliu Hațieganu” University of Medicine and Pharmacy, 400337 Cluj-Napoca, Romania; 4 Faculty of Medicine and Pharmacy, University of Oradea, 410068 Oradea, Romania; 5 Clinical Emergency County Hospital, 410039 Oradea, Romania; 6 Department of Dermatology, Faculty of Medicine, “Lucian Blaga” University of Sibiu, 550169 Sibiu, Romania; 7 Clinic of Dermatology, County Emergency Hospital Sibiu, 550245 Sibiu, Romania; 8 Santomar Oncodiagnostic Laboratory, 400350 Cluj-Napoca, Romania; 9 Department of Plastic and Reconstructive Surgery, “Prof Dr. I. Chiricuță” Institute of Oncology, 400015 Cluj-Napoca, Romania

**Keywords:** melanoma, COVID-19, diagnosis, delay, prognosis

## Abstract

The COVID-19 pandemic affected the healthcare system in our country and led non-COVID patients to postpone medical visits that were not urgent. The purpose of this study was to investigate the impact of the first year of the COVID-19 pandemic on the trends in melanoma diagnosis and to compare the pathological characteristics of melanoma patients before and during the pandemic. The number of primary cutaneous melanomas diagnosed each month between 1 March 2019 and 29 February 2020 (pre-COVID-19) and between 1 March 2020 and 28 February 2021 (COVID-19) in the North-Western Region of Romania (Cluj and Bihor counties) was determined. The pathological characteristics of melanomas diagnosed in the two intervals were compared. The number of melanoma diagnoses substantially decreased during the pandemic, with 66 (−19.3%) fewer cutaneous melanomas being diagnosed in the first year of the pandemic when compared with the previous year. The tumor thickness and mitotic rate were significantly higher in cases found during the COVID-19 pandemic. Our study suggests that COVID-19 has delayed diagnosis in patients with melanoma, leading to the detection of thicker melanomas that may increase morbidity and mortality. Further studies are needed to determine the consequences of this delay on outcomes.

## 1. Introduction

Melanoma, a malignant tumor stemming from melanocytes, is the most severe form of skin cancer, with an increasing incidence in the world’s white population. It is reported as the 19th most common cancer worldwide, with an incidence rate of approximately 25 new cases per 100,000 inhabitants in Europe. Although most cases of melanoma affect the elderly, melanoma stands for the third most common cancer in adolescents and young adults [1,2,3].

The overall 5-year survival rate of melanoma is 91.8%, but it decreases dramatically to 4% when distant metastases are present. The Breslow thickness is the most important factor in melanoma prognosis, together with ulceration, mitotic rate, histological subtype, anatomic site, sentinel lymph node positivity and increased age [4].

Overall survival decreases dramatically in the case of a high Breslow index. Thus, patients with thin melanomas (Breslow thickness ≤ 1 mm) have an excellent 5-year survival rate of 92–97% and a lower risk of regional and distant metastasis, whereas ulcerated melanomas with a thickness exceeding 4 mm carry a worse prognosis, with a 53% 5-year survival rate. Thus, early diagnosis is the best method to improve melanoma prognosis, with the delay in diagnosis being associated with a major impact on life expectancy [5].

Coronavirus disease 2019 (COVID-19) was declared a “Public Health Emergency of International Concern (PHEIC)” by the WHO in March 2020, and drastic measures and lockdowns have been implemented all over the world in an attempt to contain the pandemic. Moreover, focus shifted away from non-COVID patients and medical visits were cancelled or postponed. The fear of contracting the virus made the patients reluctant to visit healthcare services, and compliance toward scheduled visits decreased [6]. These led to delays in obtaining a diagnosis, and a decline in cancer diagnosis, especially in skin cancers, was documented in different studies [7,8,9,10,11,12,13,14,15].

This study aims to investigate the impact of the first year of the COVID-19 pandemic on the trends in the diagnosis and on the characteristics associated with prognosis in melanomas diagnosed in two medical university centers from Romania (Cluj-Napoca and Oradea).

## 2. Materials and Methods

This observational, retrospective, cohort study was carried out in Cluj and Bihor counties, in the North-Western Region of Romania. Data were obtained from all histopathological services from the two counties.

The number of primary cutaneous melanomas diagnosed between 1 March 2019 and 28 February 2021 was determined. Collected data were aggregated into pre-COVID-19 (1 March 2019–29 February 2020) and COVID-19 (1 March 2020–28 February 2021). Monthly numbers of diagnosed melanomas were compared in the two periods. Data on patient characteristics included age and gender. The tumor was characterized according to the histological subtype (superficial spreading melanoma-SSM, lentigo maligna melanoma-LMM, nodular melanoma-NM, acral lentiginous melanoma-ALM, other) and other histological characteristics (the Breslow index, presence of mitosis, ulceration, vascular and neural invasion, and tumor stage [TNM edition]).

Statistical analysis was carried out using MedCalc^®^ Statistical Software version 20.014 (MedCalc Software Ltd., Ostend, Belgium; https://www.medcalc.org (accessed on 10 November 2021). Quantitative variables were expressed as median and 25th–75th percentiles. Categorical data were characterized as frequency and percentage. Comparisons between groups (Pre-COVID-19 vs. COVID-19) were performed with the Mann–Whitney-U test (age, Breslow index, mitotic count) or chi-square test (gender, histological subtype, ulceration, vascular/lymphatic invasion, neural invasion, staging). For the Mann–Whitney-U test, no relevant deviation from the assumption of symmetry was observed. A *p* value < 0.05 was considered statistically significant.

This study was approved by the Ethics Committee of “Iuliu Hațieganu” University of Medicine and Pharmacy (88/05.04.2021).

## 3. Results

During the study period, a total of 616 patients (314 females and 302 males) were diagnosed with melanoma, 341 (177 females and 164 males) in the pre-COVID period and 275 (137 females and 138 males) during the first year of the COVID-19 pandemic. There were no relevant differences in patient characteristics between the two cohorts regarding gender. The average age for the diagnosis was 59 before the pandemic, and 63 during the pandemic, the difference being statistically significant (*p* = 0.002). The number of diagnosed melanomas decreased by 19.3% (66 cases) in the first year of the pandemic. The largest decrease was observed in April 2020 (−62.5%), May 2020 (−46.4%) and June 2020 (−51.1%) (Table 1).

The characteristics of melanomas are shown in Table 2. The Breslow index median and the mitotic rate were statistically significantly higher in cases diagnosed during the COVID-19 pandemic. There were no statistically significant differences regarding histological type and vascular and neural involvement between the two groups. Ulceration was more frequent in the COVID-19 group, but the difference did not reach statistical significance (*p* = 0.053). Regarding the tumor stage, thick melanomas were statistically significantly more frequent in the first year of the COVID-19 pandemic (*p* = 0.04). We observed a statistically significantly higher number of NMs during the COVID-19 pandemic (71 (25.8%) vs. 60 (17.6%); *p* = 0.01).

## 4. Discussion

Our study found that the first year of the COVID-19 pandemic led to a decrease in melanoma diagnoses in Romania. The decrease started in April, with the largest decrease being observed between April and June 2020. The first COVID-19 case was detected in Romania on 26 February 2020, but social lockdown was introduced only after 15 March and a substantial decline in non-urgent medical visits was observed starting in April. Interestingly, in March 2020, the number of new melanoma cases was almost double compared with March 2019, opposite to other studies that documented a decrease in skin cancer diagnoses starting in March [7,13,14].

Our study found a difference regarding the prognostic factors for melanomas diagnosed during the pandemic versus pre-pandemic ones. An increased thickness (median thickness of 2.2 mm and 1.37 mm, respectively), increased mitotic rate (median rate of 4 and 3, respectively) and increased proportion of thick melanomas (58.2% and 49.6%, respectively) were observed, similar to the findings reported in other studies [8,9,14].

Ricci et al. reported a significant decrease in melanoma diagnoses in Italy in the lockdown period. On the other hand, they reported a median thickness of 0.88 mm pre-lockdown and 0.66 mm during lockdown, which might indicate that “health-conscious” people do not underestimate the severity of their lesions despite lockdown limitations [8].

However, other studies support our findings. The study conducted by Shannon AB et al. reported an increased tumor thickness in the COVID-19 era compared to the pre-COVID-19 era (1.4 mm vs. 0.87 mm) [9]. Lallas et al. found a 36.4% decrease in melanoma cases and an increase in the advanced stages during the lockdown period compared to the pre-lockdown numbers. In our study, the decrease in melanoma cases was much lower, at around 19%, but we also documented a significant increase in advanced stages [14].

In Italy, one of the countries most affected by COVID-19 in Europe, the dermatologic consultations decreased by 80–90%. Gaudi et al. compared the melanoma cases diagnosed in 12 Italian centers in different geographic locations in the 2 months immediately after the lockdown (1 May–31 July) with the same period in the previous 3 years. The study reported a 20% reduction in melanoma cases immediately after the lockdown period, the main reduction being documented in the northern and central areas, the regions that were most affected by COVID-19 [16].

A study developed in four provinces of the Veneto region in Italy documented a 12% decrease in melanoma cases during the COVID-19 period [17], while, in Turin, Italy Cariti et al. found a 32% reduction in melanoma excisions in 2020 compared to the previous years (2017–2019), with the most frequently excised type being superficial spreading melanoma (SSM), whereas, in the previous years it was melanoma in situ. Regarding the Breslow thickness, it was higher than in the previous years (the average thickness was 1.56 mm), but the difference was not statistically significant [18].

Trepanowski et al. compared the melanoma cases in the US in the pre-COVID-19 period (1 March 2019–29 February 2020) to the COVID-19 period (1 March 2020–28 February 2021) and they reported 1834 cases in the COVID-19 period and 2062 melanoma cases pre-COVID-19, with a higher relative frequency in the COVID-19 period: stage II (18.3% vs. 14.8%), stage IV (6.1% vs. 4.6%). Moreover, the thickness, number of mitoses and the proportion of nodular subtypes were increased during the COVID-19 period [19].

In Belgium, a study comparing melanoma cases during the COVID-19 period (15 March 2020–31 December 2020) with the same periods in 2018 and 2019 found no statistically significant differences regarding the proportion of invasive melanoma cases within the three periods. Although there was no statistically significant reduction in the total number of new cases during the COVID-19 period, they reported a significant increase in melanoma thickness and ulceration [20].

In Spain, Martinez-Lopez et al. compared the melanoma cases diagnosed 12 months before the lockdown (starting with 15 March 2020), and 12 months after the lockdown. They found an 18.4% reduction in melanoma diagnoses, an increased Breslow index (median thickness of 1.08 mm and 2.65 mm, respectively) and an increased mean number of mitoses (mean number of 1.40 and 3.58, respectively). Although the frequency in ulcerations increased after the onset of confinement, it did not reach statistical significance [21].

Another study conducted in France by Molinier et al. observed an 8.2% reduction in new cases in 2020 compared to 2019, and patients diagnosed during the lockdown period (17 March–12 May 2020) had a significantly higher Breslow index (2.1 mm vs. 1.5 mm) [22].

Weston et al. evaluated in situ and invasive melanomas diagnosed each year between 1 June and 15 August 2015–2020 in New York City metropolitan area and observed no statistically significant differences between pre and post-lockdown periods (102 melanomas diagnosed in 2020 vs. an average of 106 melanomas diagnosed in the same period of the previous years). However, they reported a significantly greater average tumor thickness compared to the previous years (2.04 mm vs. 0.78 mm) and increased ulceration rates (17% vs. 6%). The proportion of nodular melanomas was also higher than in previous years (30% vs. 13.8%) [23].

Moreover, compared to the study conducted by Marson et al. [13], we did not observe a higher number of melanoma cases diagnosed during the COVID-19 recovery phase (since June 2020), with fewer melanomas being diagnosed every month during the first pandemic year until February 2021.

Concerns about diagnostic delays in cancers were expressed from the beginning of the COVID-19 pandemic, and our study confirmed this fear. Unfortunately, we did not see an increase in melanoma diagnoses during the periods with lower infection rates and more relaxed social distancing measures, suggesting that a number of melanoma patients remained undiagnosed or were diagnosed with delay. The diagnostic delay, and, consecutively, the therapeutic delay may lead to melanoma patients presenting at more advanced stages, with a potential increase in mortality and a lower survival interval. 

Davis et al. compared patients with melanoma 8 months before the lockdown (August 2019–March 2020) and after the lockdown (May 2020–December 2020) in northeastern United States. They also analyzed the 2-month period before the lockdown (January–February 2020) versus 2 months after the lockdown (May–June 2020). They reported a total of 375 patients treated before the lockdown and 313 patients after the lockdown. No statistically significant delay was found between diagnosis and definitive surgery between the pre-lockdown and post-lockdown periods (34 vs. 35 days), but there was an increase in invasive melanoma cases (stage III and IV) during the post-lockdown period (27.5% of patients compared with 7.1%) [24].

Interestingly, melanoma thickness and the percentage of thick melanomas observed in our study both before and during the pandemic were higher than the ones reported by studies conducted in other countries [8,9,14]. Higher rates of advanced tumors and a lower survival were already reported in Central and Eastern Europe in previous studies [25]. Differences in the general educational status, reduced government expenditure on education and a lack of specific health education, resulting in a lower awareness among the general population and physicians, may at least partly explain the later diagnosis of advanced stages in our study. However, the overall proportion of thin melanomas observed by us is higher than the one previously reported in Romania, showing a tendency to follow Western European trends [25].

Furthermore, the patients diagnosed before the pandemic were younger than those diagnosed during the pandemic, suggesting that elderly patients were more “health-conscious”, whereas young patients were more likely to underestimate the severity of their disease. On the contrary, Lallas et al. found that the patients with melanoma were significantly younger than those before the lockdown, which might mean that the elderly might feel a greater fear of COVID-19 [14]. Cariti et al. also reported that the average age of melanoma patients during the COVID-19 lockdown was much lower than the one before the lockdown (55 vs. 60 years) [18].

Besides the late presentation, several studies have shown that COVID-19 has dramatically affected the cancer healthcare system by limiting the screening campaigns and the access of patients to treatment. The reduced access to medical care during the lockdown has dramatically affected the daily work of dermatologists, who reported a 75% reduction in their activity since the beginning of the pandemic. Moreover, more than half of them reported that they diagnosed zero melanomas during these months [26].

Hospitals have reported a significant decrease in surgeries compared to the pre-pandemic period. Postponing diagnosis and treatment may significantly affect long-term survival [3]. Conic et al. argued that the five-year overall survival was much lower in patients who were treated after more than 60 days since the diagnosis compared to patients who underwent surgery after less than 30 days, and concluded that a delay in the treatment of cutaneous melanoma of more than 90 days from diagnosis is associated with a higher risk of death [27].

A UK study developed during the COVID-19 lockdown reported a 27% to 47% weekly decrease in treated keratinocyte carcinomas. In the United States a study reported a decrease in diagnosed cutaneous melanomas, squamous cell carcinomas and basal cell carcinomas of 43.1%, 44.1% and 51.2%, respectively [13,28].

Not only were skin cancer diagnoses and treatment affected, but several studies also reported a decrease of 40% to 72% in cancer cases diagnosed during lockdown [29,30,31]. Moreover, more than two million cancer surgeries have been postponed globally, and essential treatments for cancer were delayed in 29–44% of medical centers [32,33].

The European Academy of Dermatology and Venerology (EADV) stated that dermoscopy remains the gold standard for melanoma diagnosis and that face-to-face consultations are compulsory for an accurate diagnosis [34]. However, telemedicine, an alternative to face-to-face consultations, was widely used during the COVID-19 era, and it facilitated patients’ access to medical care. A study conducted in the UK found that face-to-face consultations for melanoma dropped from 91.4% to 21.7% during lockdown, whereas virtual appointments increased from 0.6% to 32.6% [35]. In Belgium, the Belgian Association for Dermato-Oncology published recommendations regarding the management of dermato-oncology patients during the pandemic, and clinicians started using teledermatology systems in daily practice. This might explain why the study conducted by Gedeah et al. reported a statistically non-significant decrease in melanoma diagnoses before and after the COVID-19 period [20]. Even if a face-to-face consultation is required for an accurate diagnosis, telemedicine and teledermatology can help the clinician to reduce the number of patients who need a face-to-face consultation [36]. Additionally, because of the preventive measures adopted, the screening campaigns against melanoma have been seriously affected during the pandemic. Villain et al. proposed “an alternative model” of screening campaigns using telemedicine services. The “alternative model’ should include: patients’ education through media channels, leaflets, free phone applications and free consultations [37].

To our knowledge, this is the first study in Romania to compare the trends in melanoma diagnosis and melanoma characteristics during the first year of the pandemic and the year that preceded the outbreak. The main strength of our study is the homogeneous data collection over a longer period of time compared with most of the studies on this subject. The limitation is given by the small number of institutions involved, where larger studies are needed in order to better define the impact of the pandemic on melanoma care nationally.

## 5. Conclusions

In conclusion, our findings suggest that COVID-19 has significantly delayed melanoma diagnosis and management, leading to the detection of the tumor at more advanced stages, when they are more difficult to treat, potentially leading to a higher mortality, lower survival rates and higher costs for the healthcare system. At the same time, skin cancer screening and follow up during the COVID-19 pandemic make patients more exposed to the virus and more susceptible to complications. Further studies should assess the impact of this delay on the morbidity and mortality of melanoma.

## Figures and Tables

**Table 1:** Percentage change in melanoma diagnosis by month (March 2019–February 2021).

Month	Pre-COVID-19 n (%)	COVID-19 n (%)	Change n (%)
March	21 (6.2%)	40 (14.5%)	+19 (+90.4%)
April	32 (9.4%)	12 (4.4%)	−20 (−62.5%)
May	28 (8.2%)	15 (5.5%)	−13 (−46.4%)
June	43 (12.6%)	21 (7.6%)	−22 (−51.1%)
July	34 (10.0%)	30 (10.9%)	−4 (−11.7%)
August	21 (6.2%)	18 (6.5%)	−3 (−14.2%)
September	25 (7.3%)	23 (8.4%)	−2 (−8%)
October	37 (10.9%)	26 (9.5%)	−11 (−29.7%)
November	26 (7.6%)	23 (8.4%)	−3 (−11.5%)
December	30 (8.8%)	21 (7.6%)	−9 (−30%)
January	19 (5.6%)	17 (6.2%)	−2 (−10.5%)
February	25 (7.3%)	29 (10.5%)	+ 4 (−16%)
Total	341 (100.0%)	275 (100.0%)	−66 (−19.3%)

**Table 2:** Tumor characteristics in the two groups.

Characteristics		Pre-COVID-19	COVID-19	*p*-Value
Median Breslow index (mm)(SD)		1.37 (0.5–3.5)	2.20 (0.7–5.11)	0.004
Median mitotic count (/mm^2^)(SD)		3 (1–7)	4 (1–10)	0.002
Histological subtype (n%)				
SSM		258 (75.7%)	188 (68.4%)	0.126
NM		60 (17.6%)	71 (25.8%)	
LMM		11 (3.2%)	6 (2.2%)	
ALM		8 (2.3%)	5 (1.8%)	
Other types		4 (1.2%)	5 (1.8%)	
Ulceration (n%)	No	229 (67.2%)	163 (59.3%)	0.053
	Yes	112 (32.8%)	112 (40.7%)	
Vascular/lymphatic invasion (n%)	No	324 (95%)	254 (92.4%)	0.234
	Yes	17 (5%)	21 (7.6%)	
Neural invasion (n%)	No	332 (97.4%)	269 (97.8%)	0.918
	Yes	9 (2.6%)	6 (2.2%)	
pT staging (n%)				
Tis		59 (17.3%)	39 (14.2%)	0.036
T1a		86 (25.2%)	56 (20.4%)	
T1b		26 (7.6%)	15 (5.5%)	
T2a		34 (10.0%)	23 (8.4%)	
T2b		12 (3.5%)	12 (4.4%)	
T3a		21 (6.2%)	18 (6.5%)	
T3b		39 (11.4%)	24 (8.7%)	
T4a T4b		11 (3.2%)	13 (4.7%)	
		52 (15.2%)	70 (25.5%)	
Not specified		1 (0.3%)	5 (1.8%)	
pT staging group (n%)	Thin melanoma (Tis, T1)	172 (50.4%)	115 (41.8%)	0.04
	Thick melanoma (T2, T3, T4)	169 (49.6%)	160 (58.2%)	
Total		341 (100%)	275 (100%)	

SSM—superficial spreading melanoma, NM—nodular melanoma, LMM—lentigo maligna melanoma, ALM—acral lentiginous melanoma.

## Data Availability

All data are available upon request from the correspondence author.
